# Nitrous Oxide Gas Experiments

**Published:** 1872-05

**Authors:** S. P. Cutler


### ARTICLE II.

### *Nitrous Oxide Gas Experiments.*

BY S. P. CUTLER, M.D., D.D.S.

About the middle of May, 1871, I commenced experiments to see whether or not fresh meat would spoil in the gas, if so, to ascertain whether or not, it would spoil in a longer or shorter time than in air. I first suspended about an ounce of fresh beef in a bell jar, filled the jar with gas, and set it in a deep plate, containing water to prevent any escape of gas, or any ingress of air.

I then suspended a similar piece of meat under another jar, over water same as the first, only air being in the latter jar, and let them remain undisturbed for 53 hours, the thermometer being about 72° on an average during the time.

When I examined the meat, I found both pieces badly spoiled or tainted, the piece in the jar of air, more so, than the other. On the piece in the air there was a white frosted mould standing out from the meat like fibres of cotton, an inch in length and none on the piece in the gas.

I left a piece of meat on top of one of the jars the same length of time, which was not as badly tainted as either of the others.

### SECOND EXPERIMENT.

On the last day of May, I repeated my experiments, letting the meat remain just 24 hours hours, the temperature 90° during the day, and 85° during the night, or thereabouts. On examination I found both pieces of meat badly spoiled, that in the air rather more so, though not as badly spoiled as in the first experiment, the time shorter and weather hotter.

Now, what have these experiments amounted to, or what inductions drawn therefrom? One fact is established, that is, that fresh meat spoils in the gas, though not to the same extent or with the facility, as in air.

The great and fundamental fact sought by the foregoing experiments, is, whether or not the gas actually does undergo decomposition, while in the body, either in whole or in part. The inferences from these experiments do no not necessarily militate against decomposition.

### RESULT OF OXIDATION.

These experiments have demonstrated the fact that oxidation of fresh meat does take place in the gas, but not as readily as in air, and when the meat had remained in respectively 24 and 53 hours; the gas at the same time being more than double as rich as oxygen or air. Air being in a state of mixture, gas being chemically combined, the affinities of the molecules being stronger than those of nitric acid, from which the gas is in part directed.

From the fact of the strong molecular affinity of the gas, mainly depends its safety as an anæsthetic agent in our hands; otherwise we might expect our patients to be literally eaten up or burnt up with gas; so far as the gas penetrated. From this fact, that as any gaseous compound chemically combined, is broken up or decomposed, the elements set free, are in a nascent condition, the oxygen in the case of nitrous oxide, would be corrected into *ozone*, or nascent oxygen, which possesses energetic and powerful activity, rapidly oxidizing all oxidizable agents, reoxidizing already saturated oxides.

The experiments have demonstrated that the carbon and hydrogen of the meat, do not take oxygen as readily from the gaseous compound as from the air, owing to the greater molecular affinities of the gaseous molecules, parting with the oxygen less rapidly than the air.

From the above experiments it would be but reasonable to conclude: That as the gas is inhaled into the lungs and entering the circulation, that decomposition of the gas and oxidation of the ternary elements goes on, if at all, in a slower manner, and as the per cent. of normal oxidation is diminished, just in proportion to the diminution, does anæsthesia take place.

To make my ideas more tangible, I believe anæsthesia to be dependent on deficient oxidation throughout the body, where the anæsthesia is general or local; where the anæsthetic effect is local, as in case of freezing a part by ether spray, or by salt and ice, or snow.

Any process, whether mechanical, medicinal or chemical, that diminishes oxidation in the tissues, lowers or lessens sensibility, just in proportion to the diminished oxidation. Mechanical pressure over an artery, running to a part on the carotids, running to the brain ; pressure on a nerve, running to any part, or intense cold ; all may be cited as examples of retarding oxidation, and in proportion sensibility. Any vapor or gas that replaces oxygen in the circulation, such as alcohol, taken into the stomach, there entering the circulation unchanged. Any respirable gases that take the place of oxygen in the lungs and blood, without restoring oxygen and oxidation, act as anæsthetics.

Narcotics taken into the stomach and acting on the brain, lessens oxidation and sensibility, producing narcotism. Severe shocks to the brain and nervous system lessens all the animal powers, including sensibility, so far as they go to produce anæsthesia.

THIRD EXPERIMENT.

Some time in December, 1871, I put a live mouse into a jar of pure nitrous oxide gas, I filled a half gallon jar with the gas over a little water in a deep plate; the mouse was introduced through the water, then just enough water left in the plate to prevent air from entering.

At first for about five minutes the animal appeared very much excited, and tried to climb up the sides of the jar. At seven minutes I timed its respirations, which were about 200 per minute near as I could count; at twelve minutes they were 150 per minute; at seventeen minutes they were 80; and at twenty they were 64. From that time they gradually became slower but regular until it died.

After living in the gas about thirty minutes, the mouse shook as if it had an ague for a few minutes, then became calm, sitting on its haunches with its nose resting against the side of the jar, and remained in this condition for over an hour, then settled down from exhaustion on its breast, and remained so until it died, two hours and twenty-five minutes after being introduced.

FOURTH EXPERIMENT; A MOUSE IN CHLOROFORM VAPOR.

I put a little chloroform into a large mouthed jar with paper in the bottom to keep the animal from contact, after standing uncorked for about five minutes, a sufficient time to allow the air to be removed, I let in the mouse; in five seconds it fell over on its back, and in one and a half minutes it was dead. I did not accurately note its respirations, which were very slow from the first. There were no signs of exhilaration from the first, instead, profound sedation, with regular respiration, no gasping for breath.

FIFTH EXPERIMENT; A MOUSE IN ETHER VAPOR.

I prepared the ether vapor in the same way as the chloroform vapor, then introduced a mouse and corked the bottle. At first the animal ran around on the paper for nearly a minute, then laid down on its belly and became still; the respirations now were very rapid, about 200 per minute, or as fast as I could count, for two minutes, then rapidly subsided to 40 in a minute, expiring with sudden shock and labored efforts with long intervals between, which continued two minutes, when it ceased breathing altogether, accompanied for a moment at the last with a tremor of one of the hind legs.

In both the latter experiments there may have been some air still in the bottle mixed with the vapors; not so with the nitrous oxide.

SIXTH EXPERIMENT; MOUSE IN CHLOROFORM AND ETHER VAPOR.

I put a mouse into the above vapors, and at first its breathing became slow and regular, for half a minute, then suddenly became much slower, about 50 a minute. In one minute it ceased breathing, did not moan after let into the jar; not the slightest signs of exhilaration followed. It will be inferred from the foregoing experiments that the combined vapors are more deadly than either separately; the eyes remaining wide open in all the above experiments and an unusual expression of them.

SEVENTH EXPERIMENT.

I supported a piece of fresh meat in a jar of chloroform vapor and let it remain in five days, at which time it was perfectly pure and sweet, the weather ranging about 60° or 65° in the room, at the time. I had anticipated the result before making the experiment, as there was no oxygen there to combine with the carbon and hydrogen of the meat, and no affinities strong enough between the vapor and the elements of the meat to break up the affinities of the vapor. The meat was much dryer when taken out, still there are no affinities between the vapor and the moisture of the meat. Twenty-four hours after removal of the meat it became quite dry, and had the flavor of ordinary dried meat, with still a slight smell of chloroform; no taint whatever.

May we not look upon all anæsthetics as anti "ozoneizers ?"—in other words, they prevent oxidation from taking place by their presence in the blood ; as it is well known to scientific men that oxygen, before combining with any other body, must be rendered nascent, which means that it is converted into ozone; as it never oxidises as simple oxygen, as that is inert under all circumstances.

I may in this place "barely hint" that nervous influence is an "ozoneizer," and I might go still further and hint at the idea that even realition is an ozoneizer, by sending nervous influence into the muscles, ozoneizing the oxygen in the muscles instantaneously, causing instantaneous oxidation and in consequence muscular contraction, hence ozone may be regarded as a chief factor in muscular contractility. If then all the anæsthetics arrest muscular motion and sensation, as their chief physiological and therapeutical effects, may they not be regarded as ozoneizers per se ?

The peculiar effects of nitrous oxide in arresting motion and sensation, and not consciousness entirely, is closely allied to catalepsey, if not identical with it. In that disease there is consciousness and understanding, and a comprehension of what is said to them, and what is going on around them, at the same time there is a total inability to move or offer any resistance, if harm is apprehended. During this condition, any surgical operation might be performed without any pain so long as the spell lasted. In this disease all sensorial and mater neural transmission is suspended, at the same time circulation, breathing, and consciousness are retained to a certain extent. Such is the case when the gas is given, and to a certain extent when any other similar agent is used if not carried too far.

### EIGHTH EXPERIMENT ; BREATHING OVER REPEATEDLY THE SAME AIR.

I filled a seven gallon gas bag with air, and induced a friend to inhale it in the same manner as in administering gas, which was continued two minutes. The effects on the system, were a gradual reduction of pulse seven or eight beats per minute, at the end of second minute eyes suflused but no blueness of lips. The after effects were very unpleasant, such as stupor, and fullness in the head, for several hours, with some gastric disturbance; said his feelings were like being in a close or confined place.

This single experiment does not amount to much in itself, as many similar ones should be made. These effects may be worth knowing.

(*To be Continued.*)

